# Increased photosynthesis and grain yields in maize grown with less irrigation water combined with density adjustment in semiarid regions

**DOI:** 10.7717/peerj.9959

**Published:** 2020-10-06

**Authors:** Donghua Liu, Qianmin Jia, Juan Li, Peng Zhang, Xiaolong Ren, Zhikuan Jia

**Affiliations:** 1 College of Agronomy, Northwest A&F University, Yingling, China; 2 Institute of Water Saving Agriculture in Arid Areas of China, Northwest A&F University, Yingling, China; 3 Key Laboratory of Crop Physi-ecology and Tillage Science in North-western Loess Plateau, Ministry of Agriculture, Northwest A&F University, Yingling, China; 4 State Key Laboratory of Grassland Agro-Ecosystems, Key Laboratory of Grassland Livestock Industry Innovation, Ministry of Agriculture, China, College of Pastoral Agriculture Science and Technology, Lanzhou University, Lanzhou, China

**Keywords:** Rainfall harvesting, Net photosynthetic rate, Supplemental irrigation, Climate change, Density

## Abstract

In order to design a water-saving and high-yield maize planting model suitable for semiarid areas, we conducted trials by combining supplementary irrigation with different planting densities. Three planting densities (L: 52,500, M: 75,000, and H: 97,500 plants ha^–1^) and four supplementary irrigation modes (NI: no irrigation; IV: 375 m^3^ ha^–1^ during the 11-leaf stage; IS: 375 m^3^ ha^–1^ in the silking stage; and IVS: 375 m^3^ ha^–1^ during both stages) were tested. The irrigation treatments significantly increased the leaf relative water content, but the high planting density significantly decreased the relative water content during the silking and filling stages. After supplementary irrigation during the 11-leaf stage, IV and IVS significantly increased the photosynthetic capacity, but decreased the leaf water use efficiency. IS and IVS significantly increased the photosynthetic capacity after supplementary irrigation in the silking stage over two years. During the filling stage, IV, IS, and IVS increased the two-year average net photosynthetic rate by 17.0%, 27.2%, and 30.3%, respectively. The intercellular CO_2_ concentration increased as the density increased, whereas the stomatal conductance, transpiration rate, net photosynthetic rate, and leaf water use efficiency decreased, and the high planting density significantly reduced the leaf photosynthetic capacity. The highest grain yield was obtained using the IVS treatment under the medium planting density, but it did not differ significantly from that with the IS treatment. Furthermore, the IVS treatment used two times more water than the IS treatment. Thus, the medium planting density combined with supplementary irrigation during the silking stage was identified as a suitable water-saving planting model to improve the photosynthetic capacity and grain yield, and to cope with drought and water shortages in semiarid regions.

## Introduction

Water resources are severely deficient in the arid and semiarid regions of northwestern China (Çakir, 2004; Du et al., 2010), and thus groundwater has become a major source of agricultural irrigation water in the region. However, the extensive use of groundwater resources has led to serious consequences, such as the continued decline in groundwater levels, reduced vegetation area, soil salinization, and desertification (Kang & Zhang, 2004; Wang et al., 2012). The availability of water resources will decline under climate change (Dai et al., 2010), thereby posing a huge challenge to food security in the region (Kang, 2017). Therefore, there is an urgent need to adopt water-saving irrigation technologies to use rainfall resources in a rational manner and increase crop yields in the region.

The ridge and furrow rainfall harvesting system (RFS) is a water-collecting agricultural technique, which involves constructing ridges spaced at a set distance within the field, covering the ridges, and then planting crops in the furrows. In RFS, the furrows and ridges are connected, so precipitation that runs off from the surface of the ridge is collected in the furrow where it can be utilized by the crop (Wang et al., 2014). Due to its efficient rainfall collection and preservation features, RFS has become one of the main water-saving measures used for agricultural production in arid and semiarid regions. (Gan et al., 2013; Hu et al., 2014; Wang et al., 2015).

However, the low rainfall in the arid regions of northwestern China means that the rainfall collected using RFS cannot meet the requirements for further increasing maize yields. Thus, large areas of farmland still require irrigation in order to increase maize yields (Wu et al., 2015). The introduction and application of water-saving irrigation techniques is an effective approach for increasing maize yields in northwestern China (Lian et al., 2016). Supplementary irrigation is an important agricultural water-saving measure because it can provide the appropriate amount of water during critical crop growth stages, although the water stress in stages where crops require less water to promote root growth are not considered (Xue et al., 2003; Eapen et al., 2005). Supplementary irrigation enhances the ability of crops to withstand drought (Potters et al., 2007; Zhou et al., 2009), and ultimately increases the grain yield and water use efficiency (Fabeiro, Olalla & Juan, 2001; Du et al., 2010).

Photosynthesis is the basis of maize crop development and more than 80% of the dry matter in maize comes from photosynthesis (Echarte, Rothstein & Tollenaar, 2008; Morot-Gaudry, 1986). The planting density significantly affects the canopy structure and photosynthetic rate in maize (Li & Li, 2004), as well as affecting the accumulation and distribution of dry matter (Echarte & Andrade, 2003; Sarlangue et al., 2007). At present, the key measure used to obtain maize high yields in large areas is to increase the planting density, which is a relatively simple method (Ren, Sun & Wang, 2016). Increasing the plant density maximizes the use of light, water, and heat to achieve the maximum grain yield per unit area (Ren et al., 2017; Timlin et al., 2014). Many studies suggest that increasing the planting density within a certain range can significantly increase the leaf area index and dry matter accumulation, thereby achieving high maize yields (Ren et al., 2017; Li & Li, 2004). However, an excessive planting density can lead to growth depression within the maize population, as well as resulting in poor ventilation, decreased light transmission, and accelerated leaf senescence (Borrás, Maddonni & Otegui, 2003), thereby reducing the net photosynthetic rate (Pn) (Lin et al., 2016). An excessively high planting density will also consume a large amount of the soil moisture, which can cause water stress and lead to various molecular, biochemical, and physiological changes in plants, thereby affecting growth and development (Zhu, Shi & Li, 2001; Farooq, Mubshar & Kadambot, 2014). For example, water stress can affect the plant leaf relative water content, leaf stomatal conductance (Gs), intercellular carbon dioxide concentration (C_i_), non-photosynthetic organ carbon partitioning, and osmotic protective compounds (Chaves, 2002), thereby inhibiting photosynthesis (Chen & Hao, 2015). Water stress also increases the anthesis-silking interval (Bolaños & Edmeades, 1996), decreases the silk growth rate, and reduces the growth time to ultimately cause drought-related abortion and reduce grain yields (Oury, Tardieu & Turc, 2016).

Numerous studies have demonstrated the success of RFS, and complementary irrigation techniques (Eldoma et al., 2016; Ali et al., 2019). However, further study is required to determine appropriate methods for combining the advantages of RFS planting with supplementary irrigation, and to determine the appropriate planting density. To address these aims, we studied the effects of different maize planting densities and supplementary irrigation treatments on the leaf water use efficiency, Ci, Pn, transpiration rate (Tr), dry matter accumulation, grain yield, and soil water storage under RFS. We elucidated the appropriate timing and amount of supplementary irrigation for maize under RFS, and the appropriate planting density.

## Materials and Methods

### Study site

**Figure 1:**
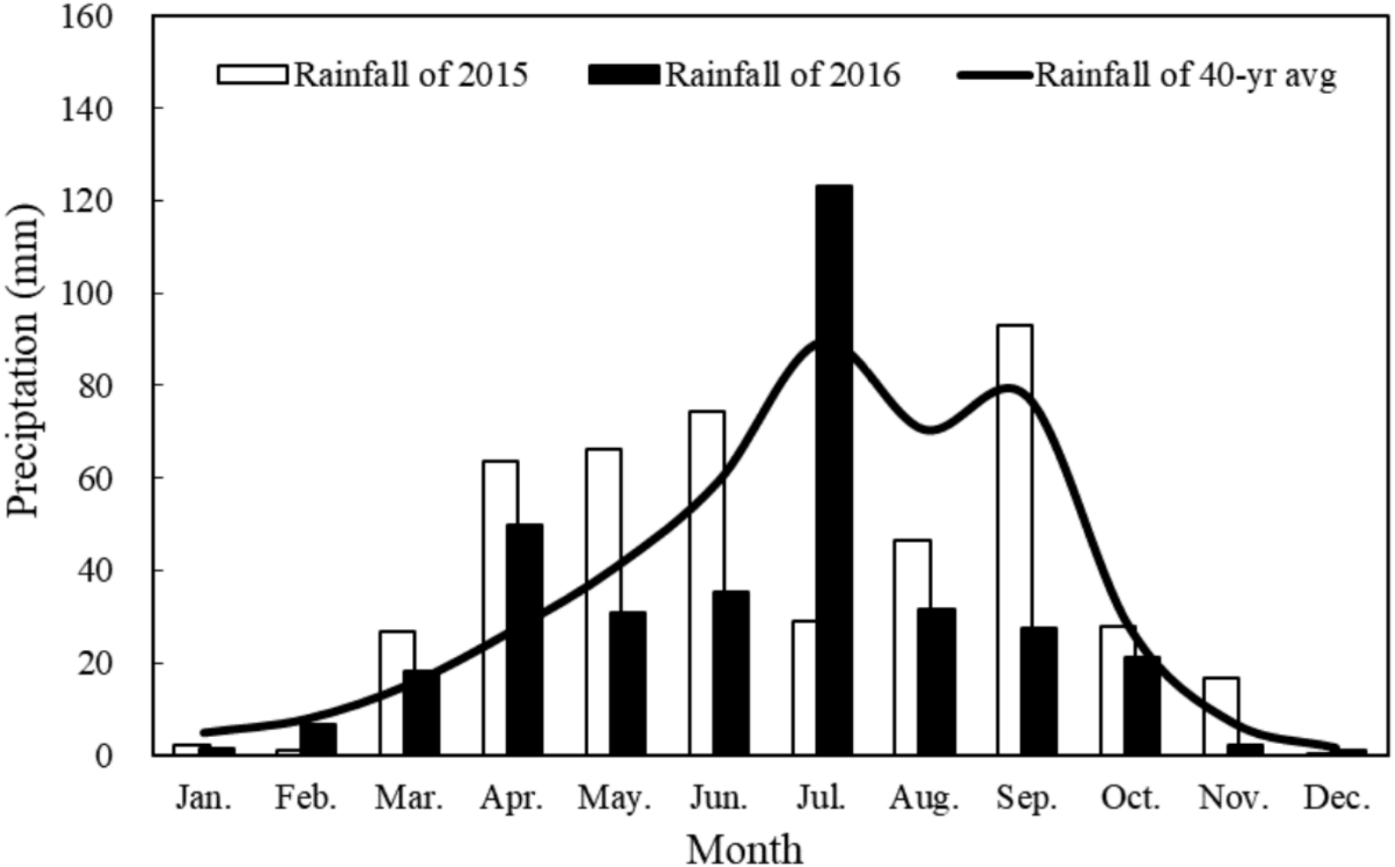
Monthly rainfall distribution in 2015 and 2016, and the 40 year average, at Pengyang Experimental Station, Ningxia Province, China.

In 2015 and 2016, field studies were performed at the Dry-Land Agricultural Experimental Station, Pengyang City, Ningxia Province, China, which is located on the Loess Plateau (35°79′N and 106°45′E, altitude of 1,800 m). The annual mean temperature is 8.1 °C. Figure 1 shows the average monthly rainfall in the test area during 2015 and 2016, as well as the 40-year average (1975–2014). Compared with the 40-year average in the growth period for maize, 2015 was a year with normal precipitation, whereas 2016 was a drought year.

#### Experimental design and field management

This study employed a randomized complete-block design. Three maize planting densities were tested under RFS with four supplementary irrigation treatments at each planting density. The three densities were low density with 52,500 plants ha^−^^1^, medium density with 75,000 plants ha^−^^1^, which is the conventional density under RFS, and high density with 97,500 plants ha^−^^1^. The four supplementary irrigation treatments comprised: NI: no irrigation; IV: 375 m^3^ ha^−^^1^ during 11-leaf stage; IS: 375 m^3^ ha^−^^1^ during the silking stage; and IVS: 375 m^3^ ha^−^^1^during both stages. The amount of irrigation used in conventional flat cropping is 1,500 m^3^ ha^−^^1^ during the maize growth period in semiarid regions, which is divided into two irrigation applications. Irrigation is applied in the furrows under RFS, and thus irrigation is only placed on the half of planting area, so the irrigation amount was halved to 375 m^3^ ha^−^^1^ per irrigation application. This study tested 12 treatments with three replicates of each. The field area for each plot was 57.6 m^2^ (length × width = 12 m × 4.8 m), and a 1.2 m wide isolation zone was set aside to prevent water leaking among the plots. In RFS, the ridges were 60 cm wide and 15 cm high, and covered with plastic (the total width of the plastic film was 0.7 m and the thickness was 0.01 mm; Gansu Tianbao Plastics Co. Ltd, China). The furrows were 60 cm wide and planted with seeds. Maize cultivar “Dafeng 30” is the main variety used locally and it is suitable for planting in semiarid areas where the active accumulated temperature ≥10 °C is above 2,700 °C, and it was sowed on April 23, 2015 and April 21, 2016. Maize was planted by hand, with a line spacing of 60 cm. The plant spacings under the low, medium, and high density treatments were 31.8 cm, 22.2 cm, and 17.1 cm, respectively.

In 2015, the experimental field was deeply plowed. The soil was prepared 10 days before planting maize and the ridges were artificially mulched. The fertilizer application was the same in all treatments, where a base fertilizer containing 150 kg ha^−^^1^ N and 150 kg ha^−^^1^ P_2_O_5_ was plowed into the topsoil in the furrows, and a topdressing fertilizer with 150 kg ha^−1^ N was applied in the 11-leaf stage. Irrigation in the 11-leaf stage was applied on July 11, 2015 and July 9, 2016, and irrigation was applied during the silking period on July 29, 2015 and July 31, 2016. Weeding and pest control were conducted. Harvesting was performed on October 10, 2015 and October 3, 2016. Maize stalks were removed after harvesting and the plots remained intact until the next year.

#### Data collection

The soil moisture contents in the 0–200 cm soil layer were measured before sowing and in the three-leaf stage, six-leaf stage, 11-leaf stage, silking stage, filling stage, wax maturity stage, and maturity stage using the soil drilling method. The soil water storage was calculated as follows (Jia et al., 2018):

\begin{eqnarray*}SWS=\sum _{i}^{n}{h}_{i}\times {\rho }_{i}\times {b}_{i}/10, \end{eqnarray*}

where *SWS* (mm) is the soil water storage, *h*_*i*_ (cm) is the thickness of a measured soil layer, *ρ*_*i*_ (g cm^−3^) is the soil bulk density in a measured soil layer, *b*_*i*_ is the soil water content in each soil layer, *n* is the number of soil layers, and *i* = 10, 20, 40 …, 200.

Leaves were sampled randomly from each plot before supplementary irrigation during the 11-leaf stage, after supplementary irrigation during the 11-leaf stage, before supplementary irrigation during the silking stage, after supplementary irrigation during the silking stage, and in the filling stage. The leaf relative water content was calculated as follows (Afzal, Duiker & Watson, 2017):

\begin{eqnarray*}RWC= \left( FW-DW \right) /(TW-DW)\times 100, \end{eqnarray*}

where *RWC* is the leaf relative water content, *FM* is the fresh leaf weight, *TM* is the weight of the leaf samples at full turgor, and *DM* is the dry weight of the leaf samples dried in an oven at 60 °C for 24 h (Jones, 2007).

Three plants were sampled randomly from each plot before supplementary irrigation during the 11-leaf stage, after supplementary irrigation during the 11-leaf stage, before supplementary irrigation during the silking stage, after supplementary irrigation during the silking stage, and in the filling stage. The leaf photosynthetic characteristics comprising Pn, Tr, C_i_, and Gs were measured using an LI-6400 portable photosynthesis system analyzer (LI-COR, Lincoln, NE, USA). The ratio of Pn relative to Tr was used as the leaf water use efficiency. Data were recorded between 9:00 and 11:00.

During the maize three-leaf stage, six-leaf stage, 11-leaf stage, silking stage, filling stage, and maturity stage, six maize plants were randomly selected from each plot and dried to constant weight at 70 °C, and the average value was used as the aboveground dry matter content of the maize in each plot. Thirty maize plants were randomly collected from each plot for drying and threshing, before measuring the grain yield, which was adjusted to a moisture content of 13%.

#### Statistical analysis

The experimental data was analysed with SPSS 13.0 (SPSS Inc., Chicago, IL, USA) and Excel 2010 (Microsoft, USA). The effects of treatments were determined using comparison of means based on the least significant difference test (LSD 0.05).

## Results

### Soil water storage

**Figure 2:**
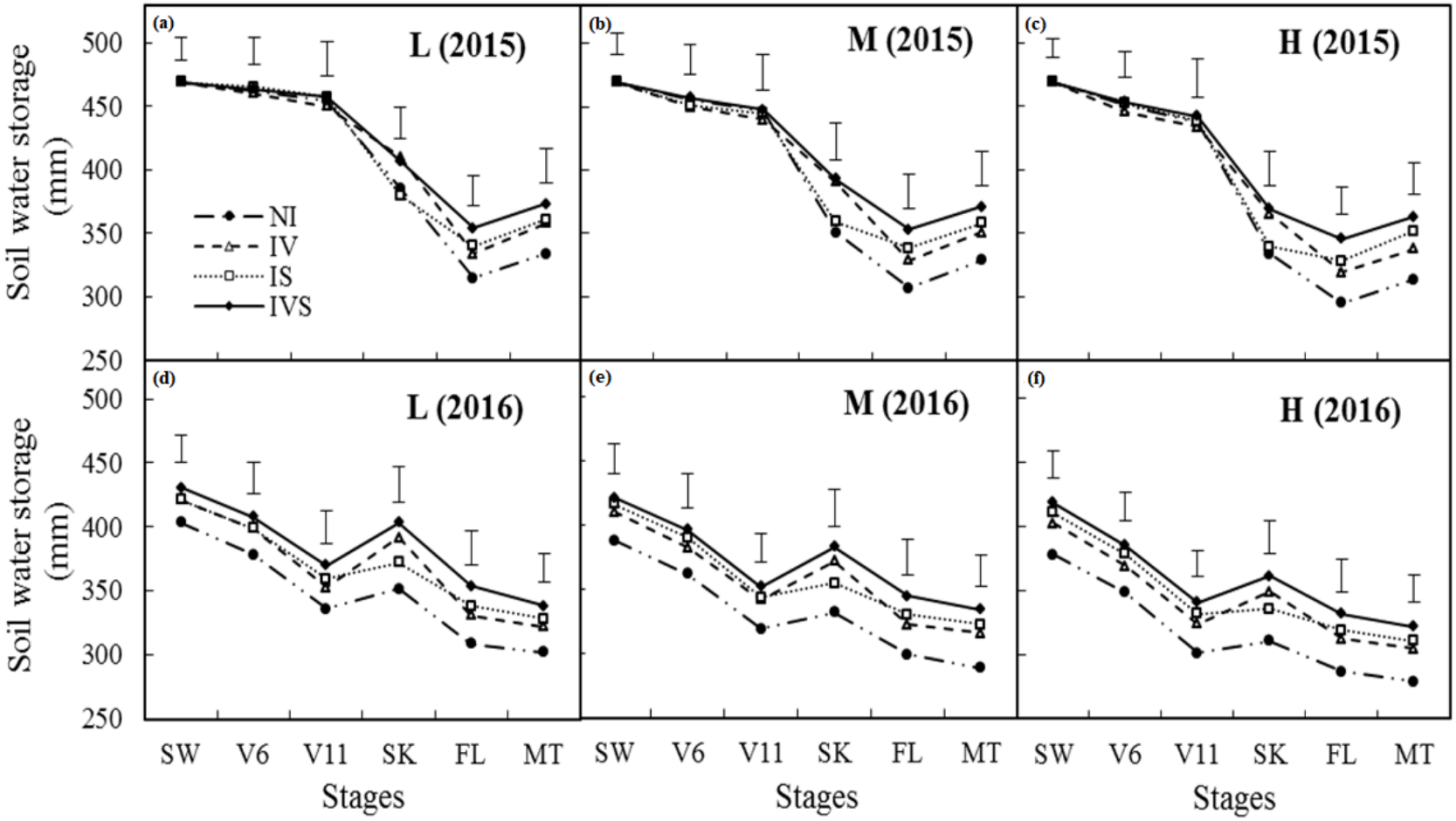
Variation in soil water storage in the 0–200 cm soil layers at six maize growth stages under different treatments in 2015 and 2016. L, low planting density (A in 2015 and D in 2016); M, medium planting density (B in 2015 and E in 2016); H, high planting density (C in 2015 and F in 2016); NI, no irrigation; IV, irrigation at the 11-leaf stage; IS, irrigation at the silking stage; IVS, irrigation at the 11-leaf and silking stages. SW, V6, V11, SK, FL and MT means sowing stage, six-leaf stage, 11-leaf stage, silking stage, filling stage, and maturity stages, respectively; Vertical bars represent the LSD values at *p* = 0.05 level.

During the normal precipitation year (2015), the difference in the soil water storage between the supplementary irrigation treatments was not significant at the sowing, six-leaf, and 11-leaf stages (Fig. 2). During the drought year (2016), the irrigation treatments significantly increased the soil water storage in the sowing, six-leaf and 11-leaf stages mainly due to the accumulated soil moisture because of the application of irrigation in 2015. The soil water storage levels were significantly higher in IV and IVS than NI during the silking stage in both years. Compared with the NI treatment, supplementary irrigation during the silking period significantly increased the soil water storage during the filling and maturity stages. We also found that the differences in the soil water storage under all three planting densities were small during the sowing, six-leaf, and 11-leaf stages in both years, but the soil water storage decreased significantly as the planting density increased during the silking, filling, and maturity stages, especially in the drought year (2016). Thus, the strong growth of maize after silking led to the consumption of a large amount of soil moisture in the high planting density treatment. The results also indicated a high level of intraspecific competitive pressure, which would be detrimental to the growth of maize during the late growth period.

### Leaf relative water content

**Figure 3:**
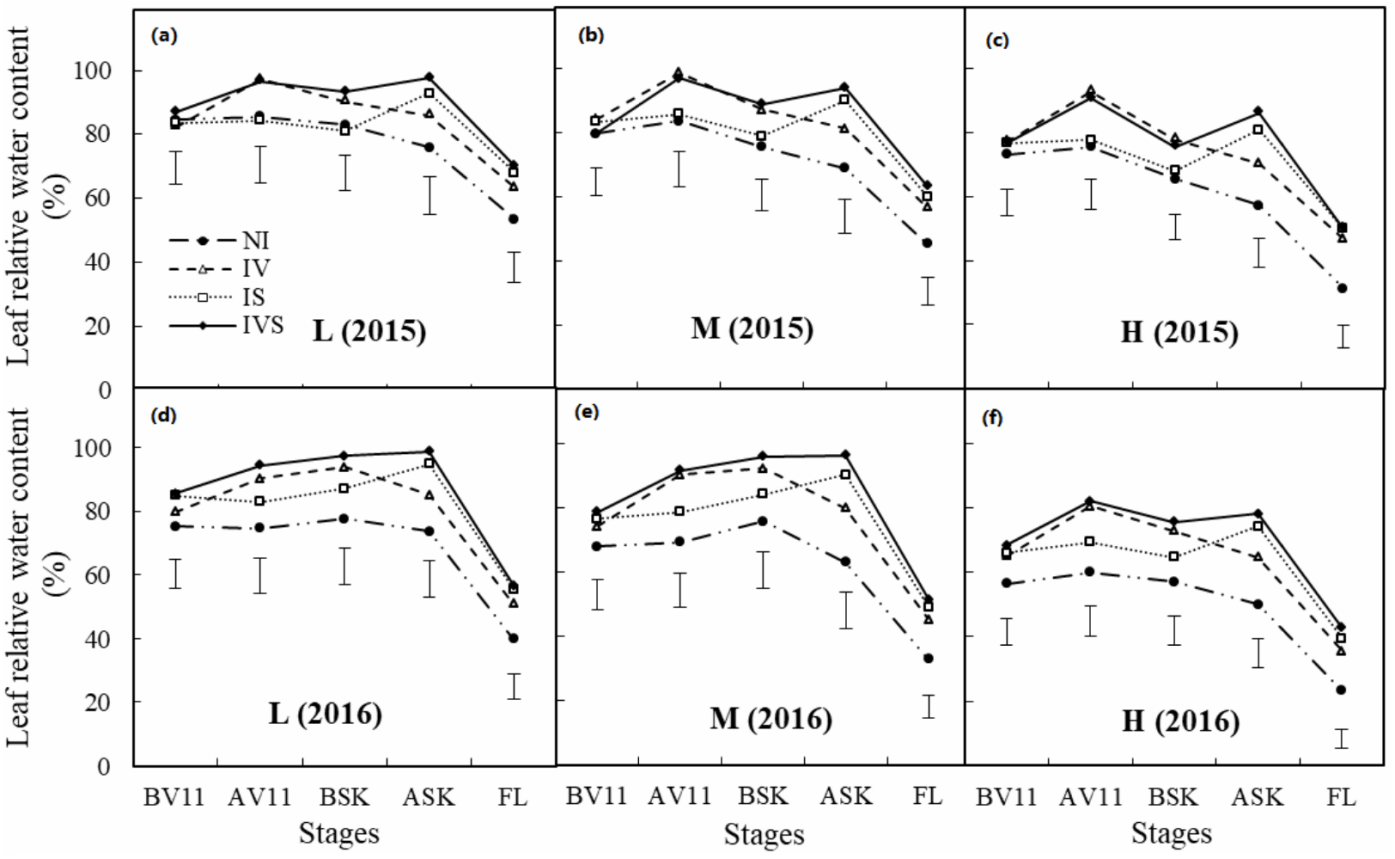
Effects of deficient treatments on leaf relative water content of maize in 2015 and 2016. L, low planting density (A in 2015 and D in 2016); M, medium planting density (B in 2015 and E in 2016); H, high planting density (C in 2015 and F in 2016); NI, no irrigation; IV, irrigation at the 11-leaf stage; IS, irrigation at the silking stage; IVS, irrigation at the 11-leaf and silking stages; BV11, AV11, BSK, ASK and FL means before supplementary irrigation at 11-leaf stage, after supplementary irrigation at 11-leaf stage, before supplementary irrigation at silking stage, after supplementary irrigation at silking stage, and supplementary irrigation at filling stage, respectively; Vertical bars represent LSD values (*P* < 0.05).

Prior to the 11-leaf stage irrigation application in 2015, there were no significant differences in the leaf relative water contents among the different irrigation levels at the same planting density (Fig. 3). However, the leaf relative water contents were significantly higher in IV and IVS than NI after the 11-leaf stage irrigation application during both years, thereby indicating that irrigating in the 11-leaf stage provided good water conditions for the vegetative growth of maize and it significantly increased the leaf relative water content.

In 2015, due to the lower rainfall from the 11-leaf stage to the silking stage, the leaf relative water contents were lower in all treatments. In 2016, two heavy rainfall events occurred between the 11-leaf stage and the silking stage, which increased the leaf relative water contents in all treatments at the low and medium planting densities. After irrigation during the silking stage, the leaf relative water contents were significantly higher under IS and IVS in both years compared with NI, and this was also the case during the filling period. Under the same irrigation mode, the leaf relative water content was significantly lower with the high planting density than the low and medium densities.

These results indicate that the leaf relative water contents were closely related to rainfall and soil moisture. Sufficient precipitation or irrigation increased the soil water storage, thereby increasing the leaf relative water content. Irrigating during the silking period increased the leaf relative water content in the filling stage, which was beneficial for filling the maize grains. However, the leaf relative water content was significantly lower under the high planting density treatment due to the greater intraspecific competitive pressure.

### Stomatal conductance (Gs) and intercellular CO_2_*concentration* (C_*i*_)

**Figure 4:**
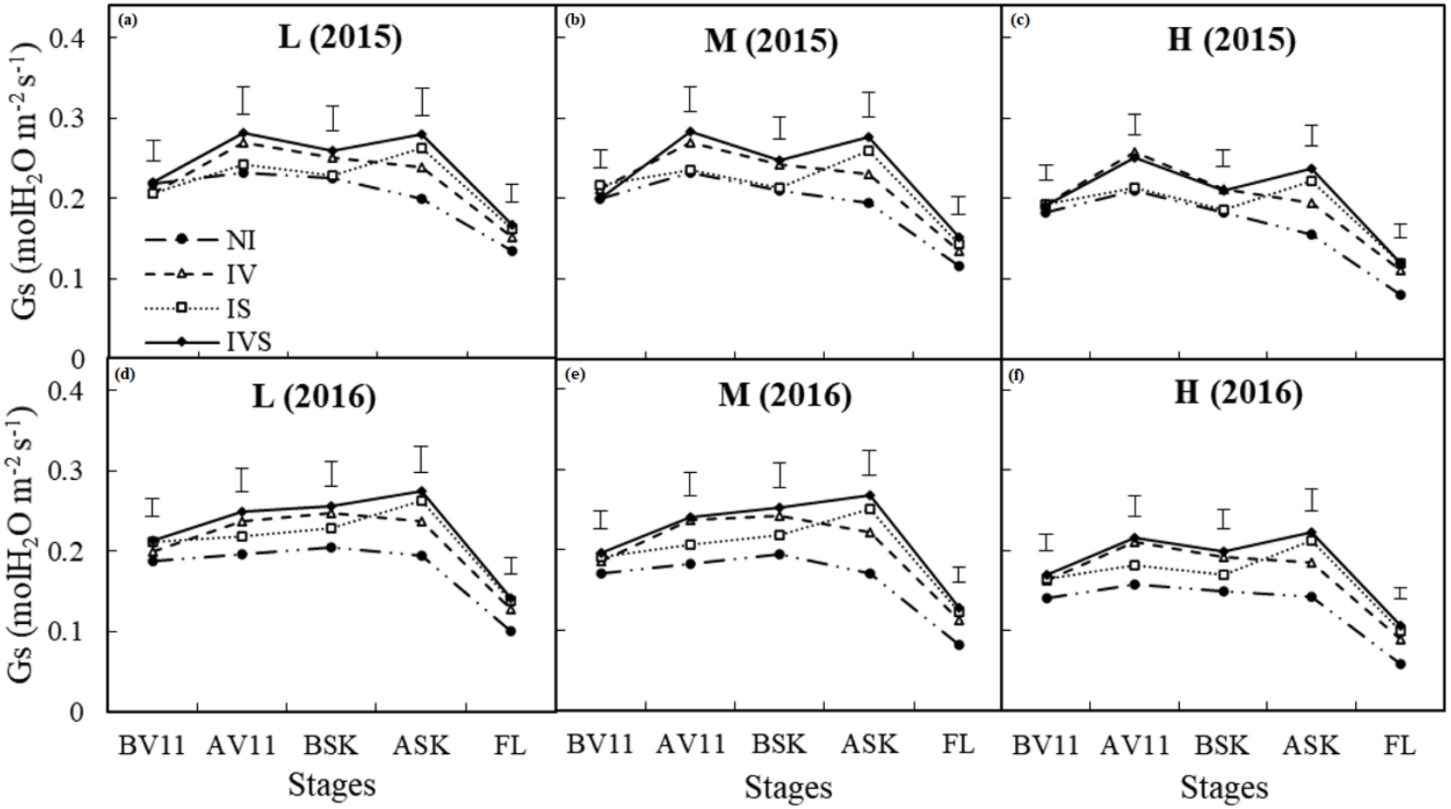
Effects of deficient treatments on stomatal conductance (Gs) of maize leaves in 2015 and 2016. L, low planting density (A in 2015 and D in 2016); M, medium planting density (B in 2015 and E in 2016); H, high planting density (C in 2015 and F in 2016); NI, no irrigation; IV, irrigation at the 11-leaf stage; IS, irrigation at the silking stage; IVS, irrigation at the 11-leaf and silking stages; BV11, AV11, BSK, ASK and FL means before supplementary irrigation at 11-leaf stage, after supplementary irrigation at 11-leaf stage, before supplementary irrigation at silking stage, after supplementary irrigation at silking stage, and supplementary irrigation at filling stage, respectively; Vertical bars represent LSD values (*P* < 0.05).

Prior to irrigation in the 11-leaf stage during 2015, there were no significant differences in Gs, whereas Gs was significantly higher under IVS in 2016 compared with NI (Fig. 4). Gs was significantly higher under IV and IVS compared with NI after irrigation in the 11-leaf stage during both years, and Gs was significantly lower under the high planting density treatment. In 2015, prior to irrigation during the silking stage, Gs decreased in each treatment due to the lower precipitation from the 11-leaf stage to the silking stage. After supplementary irrigation during the silking stage in both years, Gs was significantly higher under IS and IVS than NI, and Gs was significantly higher with the low and medium planting density treatments than the high density treatment until the filling stage. These results demonstrate that sufficient rainfall or irrigation could increase the leaf Gs, which is beneficial to leaf gas exchange and photosynthesis. However, a high planting density significantly reduced Gs in the maize leaves, which is not conducive to gas exchange during the reproductive growth stage.

**Figure 5:**
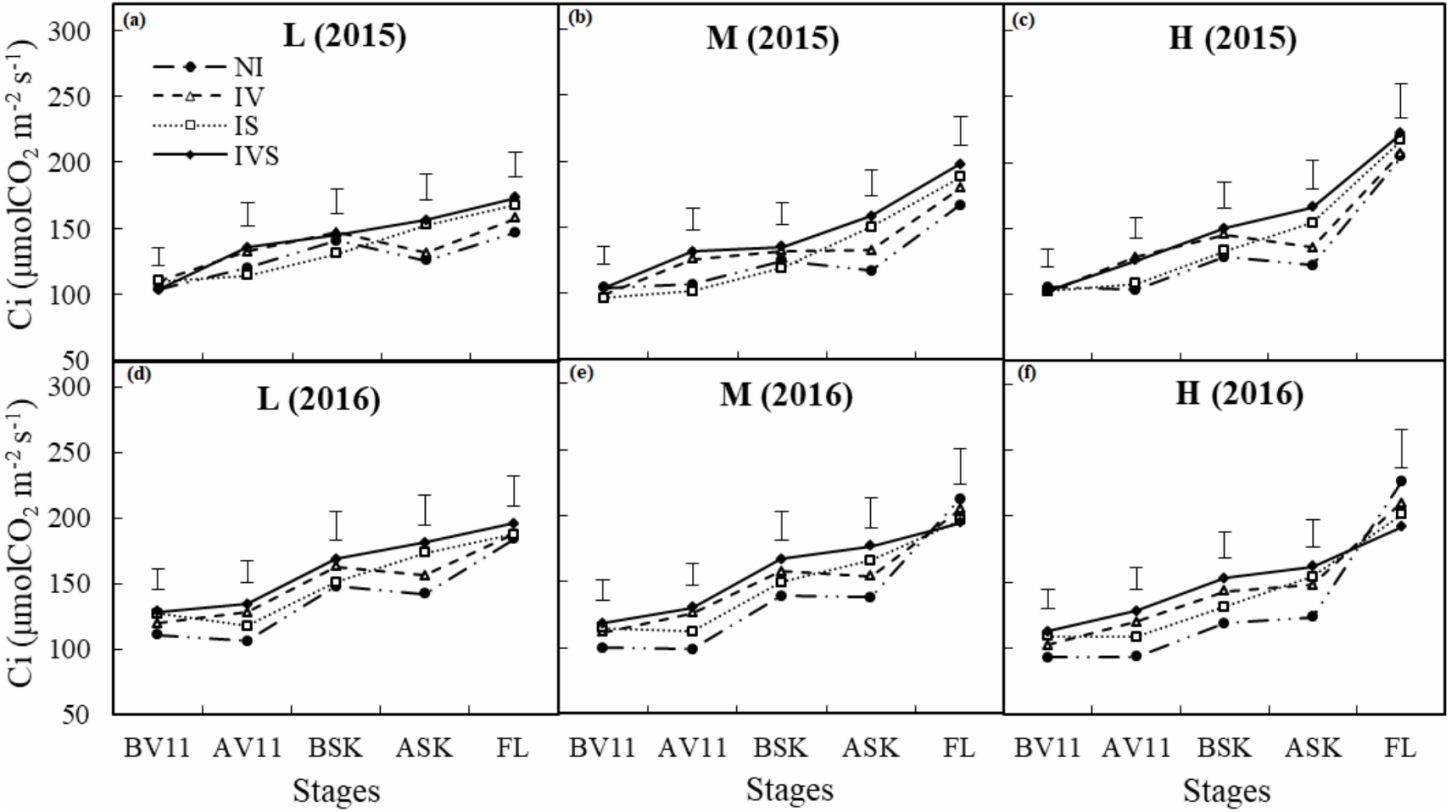
Effects of deficient treatments on intercellular CO_2_ concentration (**Ci**) of maize leaves in 2015 and 2016. L, low planting density (A in 2015 and D in 2016); M, medium planting density (B in 2015 and E in 2016); H, high planting density (C in 2015 and F in 2016); NI, no irrigation; IV, irrigation at the 11-leaf stage; IS, irrigation at the silking stage; IVS, irrigation at the 11-leaf and silking stages; BV11, AV11, BSK, ASK and FL means before supplementary irrigation at 11-leaf stage, after supplementary irrigation at 11-leaf stage, before supplementary irrigation at silking stage, after supplementary irrigation at silking stage, and supplementary irrigation at filling stage, respectively; Vertical bars represent LSD values (*P* < 0.05).

Prior to irrigation during the 11-leaf stage, the differences in C_i_ were not significant in 2015, whereas C_i_ was significantly higher under IVS than NI in 2016 (Fig. 5). After irrigation during the 11-leaf stage and silking stage, C_i_ was significantly higher under IV and IVS compared with NI in both years. These results demonstrate that supplementary irrigation could significantly increase C_i_. In the drought year (2016), plants were severely affected by drought stress in the high planting density treatment, thereby resulting in a decrease in C_i_, whereas C_i_ was affected less by the planting density in the normal year (2015). From the silking stage to the filling stage, C_i_ increased as Gs decreased in both years (Figs. 4 and 5). In 2015, C_i_ was significantly higher under IS and IVS during the filling period compared with NI at the low and medium planting densities. However, C_i_ was significantly higher under NI than IVS at the high planting density in 2016. During the filling stage, C_i_ was significantly higher at the high planting density in both years compared with the low planting density treatment with the same irrigation mode. These results may be explained by the low soil moisture damaging the leaves, thereby leading to increases in C_i_ during the filling period, especially with the high planting density in the drought year without any irrigation treatment.

### Net photosynthetic rate (Pn) and transpiration rate (Tr)

Two-way analysis of variance showed (Table 1) that prior to supplementary irrigation in the 11-leaf stage, the planting density and irrigation mode had no significant effects on Pn in 2015 (*P* > 0.05), but they had significant effects in 2016 ( *P* < 0.01). After supplementary irrigation during the 11-leaf stage, the effects of the planting density and irrigation mode on Pn were significant or highly significant in both years (*P* < 0.05 or *P* < 0.01). Compared with the mean value, the results showed that Pn decreased significantly as the planting density increased in 2016 prior to supplementary irrigation in the 11-leaf stage, whereas it increased with supplementary irrigation treatment.

**Table 1:** Effects of experimental treatments on the net photosynthetic rate of maize leaves (Pn, µmol CO_2_ m^−2^ s^−1^) in 2015–2016. Abbreviations for different treatments are defined in Fig. 3. Values followed by different letters within a column are significantly different at the 5% probability level (least significant difference; *n* = 3).

Years	Planting densities	Irrigation modes	Before irrigation at 11-leaf stage	After irrigation at 11-leaf stage	Before irrigation at silking stage	After irrigation at silking stage	Filling stage
2015	L	NI	22.5a	24.9abc	25.7ab	26.6bcd	19.8c
				IV	23.2a	28.0a	27.2a	28.2abc	22.3ab
				IS	23.2a	24.8abc	26.0ab	31.4a	23.8a
			IVS	22.8a	28.1a	27.6a	31.9a	24.0a
		M	NI	22.5a	23.5bc	24.7abc	25.2cd	18.1cd
				IV	22.7a	27.4a	26.2ab	27.1bcd	20.2bc
				IS	23.0a	23.8bc	24.7abc	30.0ab	22.6ab
			IVS	22.5a	27.8a	26.4ab	30.0ab	22.9a
		H	NI	21.9a	22.4c	21.5c	21.1e	14.4e
				IV	21.9a	25.8ab	23.0bc	23.6de	16.5de
				IS	22.7a	22.5c	21.3c	25.7cd	18.6cd
			IVS	21.8a	26.1ab	23.2bc	26.1bcd	18.6cd
		Average	L	22.9A	26.4A	26.6A	29.5A	22.5A
			M	22.7A	25.6AB	25.5A	28.0A	20.9B
			H	22.1A	24.2B	22.2B	24.1B	17.0C
			NI	22.3A	23.6B	24.0A	24.3B	17.4C
			IV	22.6A	27.0A	25.5A	26.3B	19.7B
			IS	23.0A	23.7B	24.0A	29.0A	21.7A
			IVS	22.4A	27.3A	25.7A	29.3A	21.9A
		ANOVA	PD	ns	^*^	^**^	^**^	^**^
			IM	ns	^**^	ns	^**^	^**^
		PD × IM	ns	ns	ns	ns	ns
2016	L	NI	21.4abc	22.7cde	23.3cd	23.5def	15.2de
				IV	22.7a	26.6a	26.9ab	27.2bcd	17.8bc
			IS	23.1a	24.8abc	25.2abc	29.6ab	19.5ab
			IVS	23.3a	26.7a	27.1a	31.3a	19.9a
		M	NI	19.3bcd	20.6de	21.0def	21.3fg	13.7ef
				IV	21.1abc	25.0abc	25.6abc	25.8de	16.6cd
				IS	21.8ab	23.6abcd	23.9bcd	28.6abc	17.3c
			IVS	22.5a	26.0ab	25.8abc	29.8ab	17.8bc
		H	NI	16.4e	17.7f	18.2f	18.7 g	9.2 g
				IV	18.4de	22.6cde	21.8de	22.3ef	12.3f
				IS	18.8cde	20.2ef	20.1ef	25.8de	13.3ef
			IVS	19.4bcd	23.0bcde	22.0de	26.5cd	14.6de
		Average	L	22.6A	25.2A	25.6A	27.9A	18.1A
			M	21.2B	23.8A	24.1B	26.4A	16.3B
			H	18.2C	20.9B	20.5C	23.3B	12.4C
			NI	19.0B	20.3C	20.8C	21.2C	12.7C
			IV	20.7A	24.7A	24.8A	25.1B	15.6B
			IS	21.2A	22.9B	23.0B	28.0A	16.7AB
			IVS	21.7A	25.2A	25.0A	29.2A	17.4A
		ANOVA	PD	^**^	^**^	^**^	^**^	^**^
				IM	^**^	^**^	^**^	^**^	^**^
	PD × IM		ns	ns	ns	ns	ns

**Notes.**

ANOVA, analysis of variance

** significance at 1% probability level

* significance at 5% probability level

ns not significant IM irrigation mode PD planting density

Lowercase and uppercase letters in columns indicate significant differences among planting density and irrigation mode treatments, respectively.

The average Pn was significantly higher with the low planting density treatment in both years compared with the high planting density from the 11-leaf stage to the silking stage, and the average Pn was significantly higher under IV and IVS than NI in 2016. After irrigation during the silking and filling stages, the average Pn was significantly higher with the low and medium planting densities in both years compared with the high planting density treatment, and Pn was significantly higher under IS and IVS than NI. These results indicate that the high planting density significantly reduced Pn during the maize silking and filling stages, which was not conducive to the reproductive growth of maize. Irrigating during the silking period provided water for the later growth of the maize, which was beneficial for photosynthesis during the filling stage.

The average Tr was significantly higher with the low and medium planting densities than the high planting density during the 11-leaf stage in both years (Table 2). The average Tr was significantly higher under IV and IVS than IS after irrigation during the 11-leaf stage in both years. During the silking stage, the average Tr was significantly higher with the low and medium planting densities than the high planting density in both years. After irrigation during the silking stage, the average Tr was significantly higher under IS and IVS than NI and IV. These results demonstrate that the planting density had a great influence on Tr prior to irrigation, and the high planting density significantly reduced Tr, whereas the supplementary irrigation treatments could significantly increase Tr. Comparing the average values showed that Tr was significantly higher with the low and medium planting densities than the high planting density during the filling period in both years, and the average Tr was significantly higher under IS and IVS than NI.

**Table 2:** Effects of experimental treatments on the transpiration rate of maize leaves (Tr, mmol H_2_O m^−2^ s^−1^) in 2015–2016. Abbreviations for different treatments are defined in Fig. 3. Values followed by different letters within a column are significantly different at the 5% probability level (least significant difference; *n* = 3).

Years	Planting densities	Irrigation modes	Before irrigation at 11-leaf stage	After irrigation at 11-leaf stage	Before irrigation at silking stage	After irrigation at silking stage	Filling stage
2015	L	NI	4.97abc	5.25b	4.64ab	4.72cde	4.07abc
			IV	5.21a	6.70a	4.89a	4.98cde	4.27ab
			IS	5.14ab	5.43b	4.63ab	6.07a	4.28ab
		IVS	5.15ab	6.72a	4.84ab	6.15a	4.37a
	M	NI	4.70abcd	5.12b	4.55ab	4.57de	4.00abcd
			IV	4.73abcd	6.63a	4.73ab	4.86cde	4.06abc
			IS	4.75abcd	5.20b	4.53ab	5.88ab	4.10abc
		IVS	4.79abcd	6.51a	4.75ab	5.96ab	4.15abc
	H	NI	4.35cd	5.02b	4.26ab	4.25e	3.47d
			IV	4.30d	6.23a	4.28ab	4.37e	3.61cd
			IS	4.51cd	5.11b	4.22b	5.26bcd	3.65cd
		IVS	4.45cd	6.33a	4.35ab	5.41bc	3.74bcd
	Average	L	5.12A	6.02A	4.75A	5.48A	4.25A
		M	4.74B	5.87A	4.64A	5.32A	4.08A
		H	4.40C	5.67A	4.28B	4.82B	3.62B
		NI	4.67A	5.13B	4.48A	4.51B	3.85B
		IV	4.75A	6.52A	4.63A	4.74B	3.98A B
		IS	4.80A	5.25B	4.46A	5.74A	4.01A
		IVS	4.80A	6.52A	4.65A	5.84A	4.08A
	ANOVA	PD	^**^	ns	^**^	^**^	^**^
		IM	ns	^**^	ns	^**^	^*^
		PD × IM	ns	ns	ns	ns	ns
2016	L	NI	4.23abcd	4.61de	4.04cd	4.31ef	3.33cde
				IV	4.36a	6.17a	4.95a	4.75cde	3.62abc
			IS	4.39a	4.90cd	4.20bcd	5.96a	3.86ab
			IVS	4.43a	6.12a	5.01a	6.11a	3.95a
		M	NI	4.02abcd	4.45de	4.03cd	4.18ef	3.11de
				IV	4.22abcd	5.87ab	4.69ab	4.48def	3.48bcd
				IS	4.18abcd	4.69cde	4.05cd	5.61ab	3.47bcde
			IVS	4.26abc	5.95ab	4.77ab	5.83ab	3.54abc
		H	NI	3.70d	4.05e	3.68d	3.78f	2.19f
				IV	3.89bcd	5.33bc	4.19bcd	4.14ef	2.75e
				IS	3.74cd	4.35de	3.72d	5.12bcd	2.78e
			IVS	3.83bcd	5.38bc	4.17cd	5.28bc	3.04de
		Average	L	4.35A	5.45A	4.55A	5.22A	3.69A
			M	4.17A	5.24A	4.36A	5.02A	3.40B
			H	3.79B	4.78B	3.94B	4.58B	2.69C
			NI	3.99A	4.37B	3.92B	4.09B	2.88B
			IV	4.15A	5.79A	4.58A	4.46B	3.28A
			IS	4.10A	4.65B	3.99B	5.48A	3.37A
			IVS	4.17A	5.82A	4.65A	5.74A	3.51A
		ANOVA	PD	^**^	^**^	^**^	^**^	^**^
				IM	ns	^**^	^**^	^**^	^**^
	PD × IM		ns	ns	ns	ns	ns

**Notes.**

ANOVA, analysis of variance

** significance at 1% probability level

* significance at 5% probability level; ns: not significant

IM irrigation mode PD planting density

Lowercase and uppercase letters in columns indicate significant differences among planting density and irrigation mode treatments, respectively.

### Leaf water use efficiency

Prior to supplementary irrigation during the 11-leaf stage, the leaf water use efficiency in both years was mainly affected by the planting density, where the leaf water use efficiency was significantly higher with the low and medium planting densities than the high planting density (Table 3). After supplementary irrigation during the 11-leaf stage, the leaf water use efficiency was mainly affected by the supplementary irrigation mode. The leaf water use efficiency was significantly higher under IS than IV and IVS because irrigation during the 11-leaf period significantly increased Tr, thereby decreasing the leaf water use efficiency. Similar findings were obtained during the silking period. During the filling period, compared with the high planting density, the average leaf water use efficiency in 2015 increased by 12.5% and 9.2% under the low and medium planting densities, respectively, and by 7.7% and 5.0% in 2016. Compared with NI, the IV, IS, and IVS treatments increased the average leaf water use efficiency in 2015 by 9.3%, 19.4%, and 18.3%, respectively, and by 7.5%, 12.3%, and 12.6% in 2016.

**Table 3:** Effects of experimental treatments on the water use efficiency of maize leaves (WUE_L_, µmol CO_2_ mmol H_2_O^−1^) in 2015–2016. Abbreviations for different treatments are defined in Fig. 3. Values followed by different letters within a column are significantly different at the 5% probability level (least significant difference; *n* = 3).

Years	Planting densities	Irrigation modes	Before irrigation at 11-leaf stage	After irrigation at 11-leaf stage	Before irrigation at silking stage	After irrigation at silking stage	Filling stage
2015	L	NI	4.53ab	4.73a	5.54a	5.62a	4.86abc
			IV	4.44ab	4.17ab	5.56a	5.66a	5.24ab
			IS	4.52ab	4.56ab	5.62a	5.16ab	5.56a
		IVS	4.43b	4.18ab	5.69a	5.18ab	5.49a
	M	NI	4.80ab	4.59ab	5.43a	5.51ab	4.51cd
			IV	4.80ab	4.13b	5.54a	5.57ab	4.98abc
			IS	4.84ab	4.58ab	5.44a	5.10ab	5.51a
		IVS	4.70ab	4.26ab	5.55a	5.04ab	5.53a
	H	NI	5.04ab	4.46ab	5.05a	4.96ab	4.16d
			IV	5.10a	4.13b	5.37a	5.40ab	4.57bcd
			IS	5.04ab	4.40ab	5.06a	4.88ab	5.01bc
		IVS	4.89ab	4.12b	5.32a	4.82b	4.99bc
	Average	L	4.48B	4.41A	5.60A	5.41A	5.29A
		M	4.78A	4.39A	5.49AB	5.30AB	5.13A
		H	5.02A	4.28A	5.20B	5.01B	4.70B
		NI	4.79A	4.59A	5.34A	5.36AB	4.51C
		IV	4.78A	4.15B	5.49A	5.54A	4.93B
		IS	4.80A	4.51A	5.37A	5.05B	5.39A
		IVS	4.67A	4.19B	5.52A	5.01B	5.34A
	ANOVA	PD	^**^	ns	^*^	ns	^**^
		IM	ns	^**^	ns	^*^	^**^
		PD × IM	ns	ns	ns	ns	ns
2016	L	NI	5.06ab	4.91ab	5.76ab	5.46a	4.57ab
				IV	5.23a	4.31bc	5.43abc	5.73a	4.92a
			IS	5.26a	5.07a	5.99a	5.19a	5.04a
			IVS	5.25a	4.37bc	5.42abc	5.11a	5.03a
		M	NI	4.80ab	4.64abc	5.21bc	5.09a	4.42ab
				IV	5.01ab	4.27c	5.57abc	5.76a	4.77ab
				IS	5.23a	5.03a	5.89ab	5.10a	4.99a
			IVS	5.28a	4.36bc	5.41abc	5.11a	5.02a
		H	NI	4.41b	4.36bc	4.96c	4.94a	4.20b
				IV	4.71ab	4.23c	5.21bc	5.38a	4.49ab
				IS	5.02ab	4.65abc	5.41abc	5.04a	4.78ab
			IVS	5.07ab	4.28c	5.27abc	5.02a	4.80ab
		Average	L	5.20A	4.66A	5.65A	5.37A	4.89A
			M	5.08AB	4.57A	5.52AB	5.26A	4.80AB
			H	4.80C	4.38A	5.21B	5.09A	4.57B
			NI	4.76B	4.64AB	5.31B	5.16B	4.40B
			IV	4.98AB	4.27C	5.40AB	5.62A	4.73A
			IS	5.17A	4.91A	5.76A	5.11B	4.94A
			IVS	5.20A	4.34BC	5.36AB	5.08B	4.95A
		ANOVA	PD	^*^	ns	^*^	ns	^*^
				IM	^*^	^**^	^*^	^*^	^**^
	PD × IM		ns	ns	ns	ns	ns

**Notes.**

ANOVA, analysis of variance

** significance at 1% probability level

* significance at 5% probability level

ns not significant IM irrigation mode PD planting density

Lowercase and uppercase letters in columns indicate significant differences among planting density and irrigation mode treatments, respectively.

These results indicate that severe water stress was experienced with the high planting density during the filling stage and Pn decreased in the leaves to significantly reduce the leaf water use efficiency. Supplementary irrigation during the silking stage provided favorable water conditions for filling the maize grains and, increasing the leaf Pn, which resulted in a significant increase in the leaf water use efficiency.

### Total dry matter accumulation and grain yield

In 2015 and 2016, the total dry matter accumulation increased significantly as the planting density increased (Table 4). Compared with NI, the average total dry matter accumulation in 2015 under IV, IS, and IVS increased by 7.6%, 3.3%, and 7.5%, respectively, and by 14.7%, 12.9%, and 17.4% in 2016. These results indicate that supplementary irrigation during the 11-leaf stage could increase the total dry matter accumulation, and the total dry matter accumulated in the drought year (2016) was higher than that in the normal year (2015). This effect was particularly significant in the drought year with medium and high planting densities, and during the post-silking period. The average total dry matter accumulation increased significantly as the planting density increased during the entire growing period in both years. In addition, irrigating during the silking period increased the total dry matter accumulation compared with IS and IVS during the entire growth period, and the increase was higher in the drought year than the normal year.

**Table 4:** Effects of experimental treatments on the total dry matter accumulation (TDMA, t ha^−1^) and gain yield (t ha^−1^) in 2015–2016. Abbreviations for different treatments are defined in Fig. 3). Values followed by different letters within a column are significantly different at the 5% probability level (least significant difference; *n* = 3).

Years	Planting densities	Irrigation patterns	Pre-silking TDMA	Post-silking TDMA	Entire growth period TDMA	Grain yield
2015	L	NI	11.3f	11.5d	22.8e	10.9c
			IV	11.9ef	12.4cd	24.3de	11.9bc
			IS	11.4f	13.7abc	25.1cd	12.3ab
		IVS	11.9ef	13.3abc	25.2cd	12.4ab
	M	NI	13.6de	13.1bcd	26.7bcd	11.5bc
			IV	14.7bcd	13.2bcd	27.9abcd	12.6ab
			IS	14.0cd	14.8ab	28.8abc	13.4a
		IVS	14.7bcd	14.5ab	29.2ab	13.5a
	H	NI	15.5abc	14.5ab	30.0ab	11.8bc
			IV	16.8a	14.5ab	31.4a	12.7ab
			IS	16.3ab	15.3a	31.6a	13.4a
		IVS	16.8a	15.0ab	31.8a	13.6a
	Average	L	11.6C	12.7C	24.4C	11.7B
		M	14.3B	13.9B	28.1B	12.8A
		H	16.4A	14.8A	31.2A	12.9A
		NI	13.5A	13.0C	26.5B	11.4C
		IV	14.5A	13.4BC	27.9AB	12.4B
		IS	13.9A	14.6A	28.5A	12.9A
		IVS	14.5A	14.2AB	28.7A	13.1A
	ANOVA	PD	^**^	^**^	^**^	^**^
		IM	ns	^*^	^*^	^**^
		PD × IM	ns	ns	ns	ns
2016	L	NI	10.0d	11.0e	21.0f	9.2c
				IV	11.4d	12.0cde	23.4def	11.1b
			IS	11.1d	13.4abc	24.5cde	11.3b
			IVS	11.6d	13.0abcd	24.7cde	11.4b
		M	NI	11.4d	10.8e	22.2ef	9.8c
				IV	13.4c	12.3bcd	25.7bcde	11.5b
				IS	13.3c	13.9ab	27.1abc	13.0a
			IVS	13.7bc	13.0abcd	26.7abcd	13.1a
		H	NI	13.6bc	11.4de	25.0cde	9.6c
				IV	15.3a	13.6abc	29.0ab	11.2b
				IS	15.1ab	14.7a	29.8a	12.8a
			IVS	15.7a	14.2a	29.9a	12.9a
		Average	L	11.0C	12.4B	23.4C	10.8B
			M	12.9B	12.5B	25.4B	11.9A
			H	14.9A	13.5A	28.4A	11.6A
			NI	11.7B	11.1C	22.7B	9.5C
			IV	13.4A	12.7B	26.0A	11.3B
			IS	13.2A	14.0A	27.1A	12.4A
			IVS	13.7A	13.5AB	27.1A	12.5A
		ANOVA	PD	^**^	^*^	^**^	^**^
				IM	^**^	^**^	^**^	^**^
	PD × IM		ns	ns	ns	ns

**Notes.**

ANOVA, analysis of variance

** significance at 1% probability level

* significance at 5% probability level; ns: not significant

IM irrigation mode PD planting density

Lowercase and uppercase letters in columns indicate significant differences among planting density and irrigation mode treatments, respectively.

The average grain yields in 2015 increased by 8.8% and 9.7% with the medium and high planting densities, respectively, compared with the low planting density, and similar increases of 10.2% and 8.2% were obtained in 2016. These results demonstrate that the medium and high planting densities significantly increased the grain yield, whereas the high planting density did not significantly increase the grain yield compared with the medium planting density and it even decreased the grain yield in the drought year (2016). The average grain yields in 2015 increased by 8.9%, 13.7%, and 15.2% under IV, IS, and IVS, respectively, compared with NI, and by 18.3%, 30.3%, and 31.4% in 2016. These results indicate that supplementary irrigation could significantly increase the grain yield in maize. The effect of supplementary irrigation was greater during the silking stage than the 11-leaf stage. Applying supplementary irrigation twice did not significantly increase the grain yield compared with only applying irrigation during the silking stage.

## Discussion

### Soil water content

In semiarid regions of China, crop production is severely limited by water shortages, particularly due to the mismatch between rainfall and crop water demands, and severe water shortages during the reproductive growth stage of maize (Wang et al., 2009). RFS increases the amount of rainfall infiltration and soil water storage by collecting rainfall in furrows (Ren, Jia & Chen, 2008). In the present study, we found that supplementary irrigation did not significantly increase the soil water storage prior to the silking stage in the normal year (2015), whereas it significantly increased the soil water storage in the drought year (2016) due to the lower precipitation and accumulation of water from the previous year. In addition, although the amount of irrigation used under IVS was twice that under IS, the soil water storage did not differ significantly between the two treatments. Our research area was affected by strong sunlight and strong winds, and the annual water surface evaporation was about 1,700 mm. Thus, soil water evaporation was the main cause of water losses (Feng et al., 2016; Kang et al., 2003). However, due to the limited amount of water applied in the irrigation treatment, the soil water storage did not differ significantly among the supplementary irrigation treatments.

We also found that the soil water storage did not decrease as the planting density increased before the silking stage, probably because the plants were small in this stage and the water loss was mainly caused by soil evaporation. In addition, the high planting density provided a benefit due to shading (Li et al., 2016), which reduced the sunlight reaching the surface, thereby inhibiting soil water evaporation. However, after the silking stage, the soil water storage decreased significantly with the high planting density due to the higher consumption of water for plant growth, which was disadvantageous to maize development in the later growth stages.

### Photosynthesis characteristics of maize leaves

Water stress can disrupt the condition of plants, which can regulate their metabolic and defense systems to adapt to the environment. It has been reported that the relative water content of maize leaves in the seedling stage decreased by 19.8% compared with the control after 6 days of water deficit (Nikolaeva, Maevskaya & Voronin, 2017). Studies have also shown that subjecting plants to severe water stress when the leaf relative water content was below 70% (Ennahli & Earl, 2005) could damage the photosynthetic organs in the leaves (Souza et al., 2004). The results obtained in our two-year field trial showed that the relative water content of the maize ear leaves was less than 70% during the filling stage under NI. In addition, the relative water contents were below 40% with the medium and high planting densities, which may damage the photosynthetic organs in the leaves. In both the normal year and drought year, irrigation significantly improved the leaf relative water content, especially when the irrigation was applied during the silking period, which was beneficial for photosynthesis in the maize leaves.

Pn can reflect the photosynthetic efficiency of plants. Previous studies have shown that Pn and Tr decreased in the leaves as the soil water content reduced (Rouhi et al., 2007). Similar results were found in our study. The soil water storage differed little before irrigation during the 11-leaf stage in 2015, and thus Pn did not differ significantly (Fig. 2). However, due to the accumulation of soil moisture during 2015, the supplementary irrigation treatment increased the soil water storage during the 11-leaf stage in 2016, which increased Pn, and supplementary irrigation during the silking period further increased Pn. Many studies have shown that water deficit can inhibit the photosynthetic rate in plants, mainly due to the increased stomatal resistance under drought stress limiting the diffusion of CO_2_ from the air into the leaves (Lawlor, 2002; Lavinsky et al., 2015). Water stress can limit photosynthesis due to stomatal or non-stomatal factors (Medrano et al., 2002; Flexas et al., 2006). According to Farquhar & Sharkey (1982), the decrease in Pn can be explained by an increase in stomatal resistance when Gs and C_i_ decrease simultaneously, but if Pn decreases as C_i_ increases, it is considered that the main limiting factor for Pn is the decreased photosynthetic activity of mesophyll cells.

Our results showed that Gs and C_i_ were lower under NI than the supplementary irrigation treatments, and Pn was also significantly lower after irrigation during the 11-leaf stage and silking stage. These results indicated that water stress increased the stomatal resistance of the leaves under NI, which reduced Gs and C_i_, thereby decreasing Tr and Pn during the 11-leaf stage and silking stage. However, Vitale et al. (2009) showed that maize can effectively resist drought during the vegetative growth stage and the leaf photosynthetic capacity was not affected by water stress, which appears to contradict our findings, although this disparity may be explained by differences in the degree of drought stress or the maize development stages considered.

Carvalho, Cunha & Silva (2011) found that C_i_ decreased in the early stages under water stress but increased under higher water stress. Vu et al. (1998) indicated that the RuBisCO enzyme content and activity decreased in the leaves under severe water stress conditions, which weakened the Calvin cycle and increased C_i_ in the sheath cells. Azizian & Sepaskhah (2014) found that leaf aging reduced Pn and Gs during the later growth stages in maize. According to Vitale et al. (2007), leaf gas exchange was restricted by Gs under water stress conditions, thereby leading to a decrease in Pn, but Pn increased significantly after irrigation. Our results are similar to those obtained in these previous studies. We also found that NI significantly decreased Gs, Tr, and Pn during the filling stage at the high planting density in the drought year (2016), whereas C_i_ increased. This was due to the low soil moisture content during the filling period in the drought year, which was particularly harsh with a high planting density, and this led to leaf aging and caused a decrease in Pn due to non-stomatal factors.

### Dry matter accumulation and grain yield

A previous study conducted by Fan et al. (2014) in the semiarid area of northwestern China showed that maize was more sensitive to water availability during the silking stage than the large bell stage. In the Yellow River irrigation area of Ningxia, China, Liu et al. (2012). found that the maize yields were significantly reduced by drought stress during the silking stage, which shortened the duration of the grain filling stage. By contrast, supplementary irrigation during the silking period was conducive to the accumulation of dry matter during later development and it significantly increased the grain yields. Our results also showed that supplementary irrigation significantly increased the total dry matter accumulation and grain yields compared with the un-irrigated control, but the effects of applying supplementary irrigation twice and only irrigating during silking stage did not differ.

Zhang et al. (2005) showed that when the planting density was excessively high, maize plants competed for nutrients, light, and water, thereby resulting in insufficient grain filling during the later growth stages, with reductions in the 100-grain weight, dry matter accumulation, and yields. Dou, Yu & Yu (2013) found that a medium planting density (60,000 plants to 68,000 plants ha^−1^) with supplementary irrigation improved the water consumption by maize, thereby increasing the maize dry matter accumulation and grain yield. We obtained similar results where the total dry matter accumulation increased significantly with the planting density prior to the silking stage over two years, but the total dry matter accumulation increased slowly as the planting density increased after the silking stage. These results can be explained by the low rainfall after silking and the competitive pressure increasing under high density maize planting to cause significant decreases in the leaf area index and photosynthetic rate during the filling stage. The restricted accumulation of dry matter after the silking stage under high density planting then affected grain development and the yield, especially under the conditions with no irrigation. Farnham (2001) reported that the increase in the grain yield was not significant when the maize planting density was increased from 59,000 plants ha^−1^ to 89,000 plants ha^−1^. Many studies have shown that an excessive planting density reduces the availability of growth factors such as light, heat, and moisture (Widdicombe & Thelen, 2002), thereby leading to reductions in the leaf area, aboveground dry matter, and photosynthetic products distributed to the maize ear, and ultimately to decreased grain yields (Ciampitti & Vyn, 2011; Tokatlidis & Koutroubas, 2004). Our results were similar but we also found that supplementary irrigation during the silking stage was helpful for increasing the yield at a high planting density. Using a combination of rainfall harvesting and supplementary irrigation in the semiarid areas of northwestern China, the optimal planting density for maize is 75,000 plants ha^−1^, which is higher than the traditional planting density of 60000–68000 plants ha^−1^ described by Dou, Yu & Yu (2013). This is mainly because the rainfall harvesting system provides a good hydrothermal environment for the growth of maize (Liu et al., 2014; Wu et al., 2015), thereby allowing further increases in the grain yield as the planting density increases. In our study, the medium planting density combined with the irrigation treatment during the silking stage achieved a higher average annual yield (13.2 t ha^−1^), which was about 24.3% higher than that under the medium planting density treatment without irrigation.

Water shortage is the main factor that limits food production in arid and semiarid regions, and the traditional irrigation method that utilizes water in a wasteful manner cannot be maintained in this region due to the excessive consumption of water (Deng et al., 2006). The use of RFS to reduce the amount of irrigation is potentially interesting and it should be further explored. Simple and convenient irrigation schedules are useful for local farmers, especially water-saving irrigation techniques, which have great potential for crop production in the semiarid regions of China. Our results suggest that supplementary irrigation during the silking period under RFS may be an effective water-saving method for maize cultivation in semiarid areas.

## Conclusion

Based on two years of field research, we showed that sowing 75,000 plants ha^−1^ and irrigation with 375 m^3^ ha^−^^1^ in the silking stage is a reasonable water-saving cultivation technique for maize in a semiarid area. This method can increase the soil water storage capacity, photosynthetic capacity of the maize leaves, dry matter accumulation, and grain yields in semiarid areas with annual rainfall of approximately 400 mm. Compared with conventional planting, this method allows the planting density to be increased by 43% and halves the amount of irrigation water applied. Therefore, it can be used as a water-saving planting method for maize cultivation in order to cope with drought and water shortages in semiarid regions.

##  Supplemental Information

10.7717/peerj.9959/supp-1 Data S1 Original Data

## Additional Information and Declarations

### Data Availability

The following information was supplied regarding data availability: The raw measurements are available in the Supplemental File.
